# Non-contiguous finished genome sequence of *Phocaeicola abscessus* type strain 7401987^T^

**DOI:** 10.4056/sigs.4428244

**Published:** 2013-12-15

**Authors:** Véronique Roux, Catherine Robert, Didier Raoult

**Affiliations:** 1 Aix Marseille Université, Faculté de médecine, Aix-Marseille Université, Marseille cedex, France

**Keywords:** *Corynebacterium timonense*, *Actinobacteria*

## Abstract

*Phocaeicola abscessus* strain 7401987^T^ is the sole member of the genus *Phocaeicola*. This bacterium is Gram-negative, non-spore-forming, coccoid to rod-shaped and motile by lophotrichous flagella. It was isolated from a human brain abscess sample. In this work, we describe a set of features of this organism, together with the complete genome sequence and annotation. The 2,530,616 bp long genome contains 2,090 protein-coding genes and 54 RNA genes, including 4 rRNA operons.

## Introduction

*Phocaeicola abscessus* strain 7401987^T^(CSUR P22^T^= DSM 21584^T^= CCUG 55929^T^) is the type strain of *P. abscessus*. This bacterium was isolated from a brain abscess sample from a 76-year-old patient who underwent neurosurgical intervention after cancer of the face [1]. It is a Gram-negative strictly anaerobic coccoid to rod-shaped bacterium. Currently, the genus *Phocaeicola* contains only one species [2].

Here we present a summary classification and a set of features for *P. abscessus,* together with the description of the non-contiguous finished genomic sequencing and annotation.

## Classification and features

**Figure 1:**
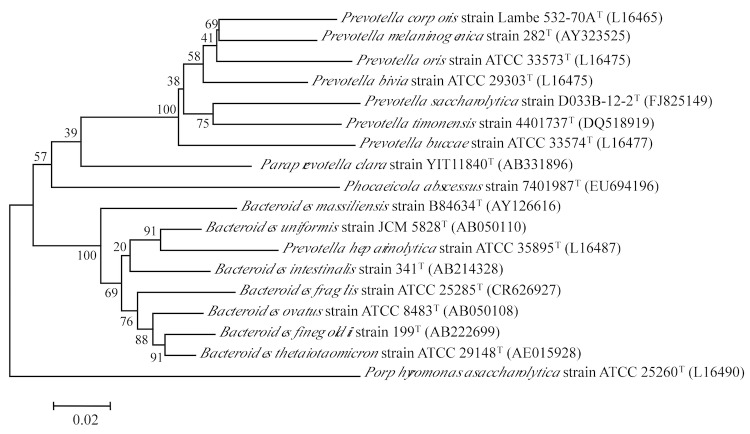
Phylogenetic tree highlighting the position of *Phocaeicola abscessus* strain 7401987^T^ relative to bacteria included in the *Prevotella, Bacteroides* and *Paraprevotella* genera by comparison of 16S rRNA gene sequences. GenBank accession numbers are indicated in parentheses. Sequences were aligned using CLUSTALX, and phylogenetic inferences obtained using the neighbor joining method within the MEGA 5 software [3]. Numbers at the nodes are percentages of bootstrap values obtained by repeating the analysis 1,000 times to generate a majority consensus tree. *Porphyromonas asaccharolytica* was used as outgroup. The scale bar represents 0.02 nucleotide change per nucleotide position.

The 16S rRNA gene sequence of *P. abscessus* strain 7401987^T^ was compared with sequences deposited in the Genbank database, confirming the initial taxonomic classification. Figure 1 shows the phylogenetic neighborhood of *P. abscessus* in a 16S rRNA based tree. The bacterium was characterized in 2007. It was isolated in the Timone Hospital microbiology laboratory (Table 1).

**Table 1:** Classification and general features of *Phocaeicola abscessus* strain 7401987^T^

**MIGS ID**	**Property**	**Term**	**Evidence code^a^**
	Current classification	Domain *Bacteria*	TAS [4]
		Phylum *Bacteroidetes*	TAS [5,6]
		Class *Bacteroidia*	TAS [5,7]
		Order *Bacteroidales*	TAS [5,8]
		Genus *Phocaeicola*	TAS [1]
		Species *Phocaeicola abscessus*	TAS [1]
		Strain 7401987^T^	TAS [1]
	Gram stain	Negative	TAS [1]
	Cell shape	Pleomorphic forms	TAS [1]
	Motility	Motile	TAS [1]
	Sporulation	Non-sporulating	TAS [1]
	Temperature range	Mesophile	TAS [1]
	Optimum temperature	37°C	TAS [1]
MIGS-6.3	Salinity	Not reported	IDA
MIGS-22	Oxygen requirement	Strictly anaerobic	TAS [1]
	Carbon source	Asaccharolytic	TAS [1]
	Energy source	Heterotrophic	NAS
MIGS-6	Habitat	Host	IDA
MIGS-15	Biotic relationship	Free living	IDA
MIGS-14	Pathogenicity Biosafety level Isolation	Unknown 2 Human brain abscess	NAS
MIGS-4	Geographic location	Marseille, France	IDA
MIGS-5	Sample collection time	2007	IDA
MIGS-4.1	Latitude	43°18 N	IDA
MIGS-4.1	Longitude	5°23 E	IDA
MIGS-4.3	Depth	Surface	IDA
MIGS-4.4	Altitude	21 m above sea level	IDA

Evidence codes - IDA: Inferred from Direct Assay; TAS: Traceable Author Statement (i.e., a direct report exists in the literature); NAS: Non-traceable Author Statement (i.e., not directly observed for the living, isolated sample, but based on a generally accepted property for the species, or anecdotal evidence). These evidence codes are from the Gene Ontology project [9]. If the evidence is IDA, then the property was directly observed for a live isolate by one of the authors or an expert mentioned in the acknowledgements.

**Figure 2:**
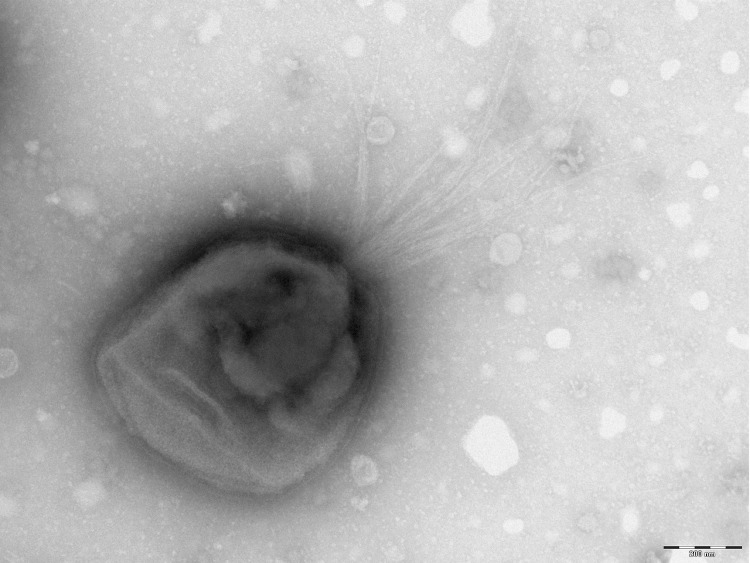
Transmission electron microscopy of *P. abscessus* strain 7401987^T^, using a Morgani 268D (Philips) at an operating voltage of 60kV. The scale bar represents 200 νm.

Cells are coccoid (0.3-0.6 μm wide and 0.4-0.9 μm long) to rod-shaped (0.4-1.7 μm wide and 1.2-6.5 μm long) and motile by flagella in a lophotrichous arrangement. Optimal growth of strain 7401987^T^ occurs at 37°C with range for growth between 30 and 37 °C. Surface colonies on chocolate agar after 7 days incubation at 37 °C under anaerobic conditions were white, circular, regular, smooth, shiny, convex and 1 mm in diameter. The isolate was asaccharolytic. Activities of acid phosphatase, naphthol-AS-BI-phosphohydrolase, N-acetyl-β-glucosaminidase, α-fucosidase, α-galactosidase, β-galactosidase, β-galactosidase 6-phosphate, α-glucosidase, N-acetyl-β-glucosaminidase, alkaline phosphatase, leucyl glycine arylamidase and alanine arylamidase were detected. The fatty acid profile was characterized by the predominance of anteiso-C_15:0_ (28.2%), C_16:0_ (18.0%), iso-C_15:0_ (12.3%) and iso-C_17:0__3-OH_ (11.7%). The size and ultrastructure of cells were determined by negative staining transmission electron microscopy. (Figure 2). Cells are coccoid (0.3-0.6 μm wide and 0.4-0.9 μm long) to rod-shaped (0.4-1.7 μm wide and 1.2-6.5 μm long).

## Genome sequencing and annotation

### Genome project history

The organism was selected for sequencing on the basis of its phylogenetic position and 16S rRNA similarity to other members of the order *Bacteroidales* and is part of study of the new species characterized in our laboratory. A summary of the project information is shown in Table 2. The EMBL accession number is CAKQ01000000 and consists of 39 contigs (≥ 500 bp) and 9 scaffolds. Table 2 shows the project information and its association with MIGS version 2.0 compliance.

**Table 2:** Project information

**MIGS ID**	**Property**	**Term**
MIGS-31	Finishing quality	High-quality draft
MIGS-28	Libraries used	One paired end 3-kb library and one Shotgun library
MIGS-29	Sequencing platforms	454 GS FLX Titanium
MIGS-31.2	Fold coverage	35.9×
MIGS-30	Assemblers	Newbler version 2.5.3
MIGS-32	Gene calling method	Prodigal
	EMBL ID	CAKQ01000000
	EMBL Date of Release	February 12, 2012
	Project relevance	Study of new species isolated in the URMITE

### Growth conditions and DNA isolation

*P. abscessus* strain 7401987^T^, was grown anaerobically on chocolate agar at 37°C. Ten petri dishes were spread and resuspended in 3 ml of TE buffer. Three hundred μl of 10% SDS and 150 μl of proteinase K were then added and incubation was performed overnight at 56°C. The DNA was then extracted using the phenol/chloroform method. The yield and the concentration was measured by the Quant-it Picogreen kit (Invitrogen) on the Genios Tecan fluorometer at 88 ng/µl.

### Genome sequencing and assembly

Shotgun and 3-kb paired-end sequencing strategies were performed. The shotgun library was constructed with 500 ng of DNA with a GS Rapid library Prep kit (Roche). For the paired-end sequencing, 5 µg of DNA was mechanically fragmented on a Hydroshear device (Digilab) with an enrichment size at 3-4 kb. The DNA fragmentation was visualized using a 2100 BioAnalyzer (Agilent) on a DNA labchip 7500 with an optimal size of 3.1 kb. The library was constructed according to the 454 GS FLX Titanium paired-end protocol. Circularization and nebulization were performed and generated a pattern with an optimal size of 579 bp. After PCR amplification through 17 cycles followed by double size selection, the single stranded paired-end library was then quantified using a Genios fluorometer (Tecan) at 8,770 pg/µL. The library concentration equivalence was calculated as 1.39E+10 molecules/µL. The library was stored at -20°C until further use.

The shotgun and paired-end libraries were clonally-amplified with 0.5 cpb and 2 cpb in 3 and 2 SV-emPCR reactions with the GS Titanium SV emPCR Kit (Lib-L) v2 (Roche). The yields of the emPCR were 9.63% and 10.3%, respectively, in the 5 to 20% range from the Roche procedure. Approximately 790,000 beads for the shotgun application and for the 3kb paired end were loaded on a GS Titanium PicoTiterPlate PTP Kit 70x75 and sequenced with a GS FLX Titanium Sequencing Kit XLR70 (Roche). The run was performed overnight and then analyzed on the cluster through the gsRunBrowser and Newbler assembler (Roche). A total of 311,276 passed filter wells were obtained and generated 35.9 Mb with a length average of 282 bp. The passed filter sequences were assembled using Newbler with 90% identity and 40 bp as overlap. The final assembly identified 9 scaffolds and 39 contigs (>500 bp).

### Genome annotation

Open Reading Frames (ORFs) were predicted using Prodigal [10] with default parameters but the predicted ORFs were excluded if they were spanning a sequencing GAP region. The predicted bacterial protein sequences were searched against the GenBank database [11] and the Clusters of Orthologous Groups (COG) databases [12] using BLASTP. The tRNAscan-SE tool [13] was used to find tRNA genes, whereas ribosomal RNAs were found by using RNAmmer [14]. Transmembrane domains and signal peptides were predicted using TMHMM [15] and SignalP [16], respectively. ORFans of alignment length greater than 80 amino acids were identified if their BLASTp *E-*value was lower than 1e-03. If alignment lengths were smaller than 80 amino acids, we used an *E*-value of 1e-05. Such parameter thresholds have been used in previous works to define ORFans.

To estimate the mean level of nucleotide sequence similarity at the genome level between *P. abscessus* and *Prevotella timonensis, Bacteroides thetaiotaomicron* and *Paraprevotella clara*, we compared the ORFs using only comparison sequences in the RAST server [17] at a query coverage of ≥70% and a minimum nucleotide length of 100 bp.

## Genome properties

**Figure 3:**
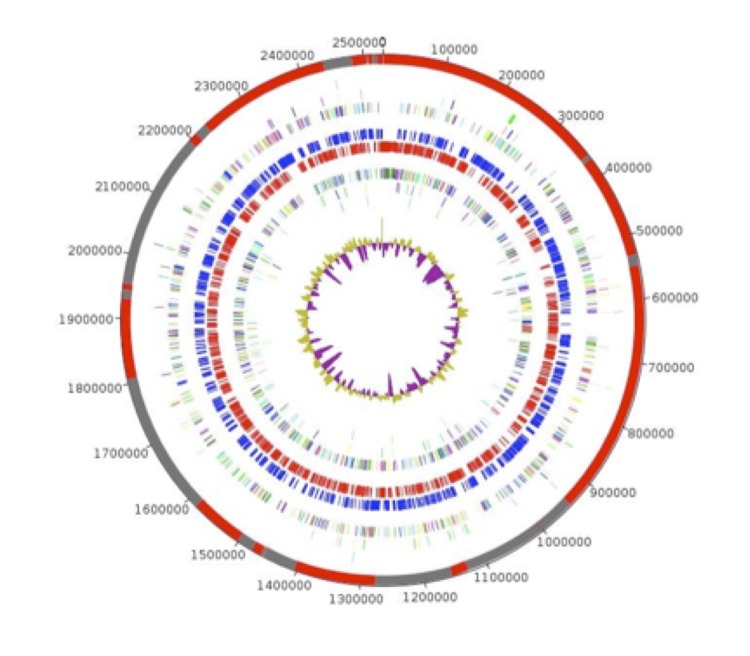
Graphical circular map of *Phocaeicola abscessus* genome. From outside to the center: Genes on the forward strand colored by COG categories (only genes assigned to COG), genes on the reverse strand colored by COG categories (only gene assigned to COG), RNA genes (tRNAs green, rRNAs red), GC content and GC skew (three circles), GC content.

The genome is 2,530,616 bp long with a 47.31% GC content (Table 3, Figure 3). Of the 2,144 predicted genes, 2,090 were protein-coding genes, and 54 were RNAs. A total of 1,464 genes (70.05%) were assigned a putative function. A total of 112 genes were identified as ORFans (5.39%). The remaining genes were annotated as hypothetical proteins (436 genes (20.86%)). The remaining genes were annotated as either hypothetical proteins or proteins of unknown functions. The distribution of genes into COGs functional categories is presented in Table 4. The properties and the statistics of the genome are summarized in Tables 3 and 4. Two CRISPRs were found using CRISPERfinder program online [18]. The first one on contig 1 includes at least 3 predicted spacer regions and the second one on contig 18 includes at least 53 predicted spacer regions.

**Table 3:** Nucleotide content and gene count levels of the genome

Attribute	Value	% of total^a^
Genome size (bp)	2,530,616	100
DNA coding region (bp)	2,284,358	90.26
DNA G+C content (bp)	1,197,056	47.31
Total genes	2,144	100
RNA genes	54	2.52
Protein-coding genes	2,090	97.48
Genes with function prediction	1,464	70.05
Genes assigned to COGs	1,433	68.56
Genes with peptide signals	554	26.51
Genes with transmembrane helices	382	18.28

a) The total is based on either the size of the genome in base pairs or the total number of protein coding genes in the annotated genome.

**Table 4:** Number of genes associated with the 25 general COG functional categories

**Code**	**Value**	**%age**	**Description**
J	141	6.75	Translation
A	0	0	RNA processing and modification
K	82	3.92	Transcription
L	103	4.93	Replication, recombination and repair
B	0	0	Chromatin structure and dynamics
D	20	0.96	Cell cycle control, mitosis and meiosis
Y	0	0	Nuclear structure
V	36	1.72	Defense mechanisms
T	50	2.39	Signal transduction mechanisms
M	143	6.84	Cell wall/membrane biogenesis
N	2	0.10	Cell motility
Z	1	0.05	Cytoskeleton
W	0	0	Extracellular structures
U	29	1.39	Intracellular trafficking and secretion
O	63	3.01	Posttranslational modification, protein turnover, chaperones
C	86	4.11	Energy production and conversion
G	119	5.69	Carbohydrate transport and metabolism
E	122	5.84	Amino acid transport and metabolism
F	52	2.49	Nucleotide transport and metabolism
H	81	3.88	Coenzyme transport and metabolism
I	47	2.25	Lipid transport and metabolism
P	89	4.26	Inorganic ion transport and metabolism
Q	20	0.96	Secondary metabolites biosynthesis, transport and catabolism
R	225	10.77	General function prediction only
S	78	3.73	Function unknown
X	657	31.44	Not in COGs

The total is based on the total number of protein coding genes in the annotated genome.

### Comparison with other genomes

*Phocaeicola abscessus* is the sole bacterium included in the genus *Phocaeicola*. We compared the genome of *P. abscessus* with those of *Prevotella timonensis* (CBQQ010000001) *Paraprevotella clara* (AFFY01000000) and *Bacteroides thetaiotaomicron* (AE015928.1). *P. abscessus* showed a mean nucleotide sequence similarity of 76.40%, 77.06% and 77.52% at the genome level (range 70-92.25%, 70.04-95.51% and 70.04-93.02%) with *P. timonensis*, *P. clara* and *B. thetaiotaomicron*, respectively. Presently, the family to which *P. abscessus* belongs is undetermined and the sole comparison based on nucleotide sequence similarity may not be sufficient to answer this question. In the future, further comparison of the genomes will allow us to find traits to classify the genus *Phocaeicola* in one of these 3 families or to create a new family, the family *Phocaeicolaceae.*
